# Decreased Serum and Salivary Levels of Aquaporin 5 in Oral Lichen Planus

**DOI:** 10.1002/cre2.70107

**Published:** 2025-03-18

**Authors:** Farzaneh Agha‐Hosseini, Yalda Elham, Iraj Mirzaei‐Dizgah, Mahdieh‐Sadat Moosavi

**Affiliations:** ^1^ Dental Research Center, Dentistry Research Institute Tehran University of Medical Sciences Tehran Iran; ^2^ Department of Oral Medicine, Faculty of Dentistry Tehran University of Medical Sciences Tehran Iran; ^3^ The Academy of Medical Sciences Tehran Iran; ^4^ Department of Physiology, Faculty of Medicine Aja University of Medical Sciences Tehran Iran

**Keywords:** aquaporin, autoimmunity, oral lichen planus, xerostomia

## Abstract

**Objectives:**

Oral lichen planus (OLP) is an autoimmune disease with unknown etiology. Many OLP patients complain of xerostomia, and studies are still ongoing to find the reason for this manifestation. Aquaporin 5 has been expressed in salivary glands, and its physiological significance in transferring water as transcellular is properly identified. In this study, for the first time, we have investigated the serum and salivary levels of aquaporin 5 (as a salivary gland biomarker) in oral lichen planus patients with xerostomia.

**Material and Methods:**

Thirty patients with OLP and xerostomia and 30 healthy individuals were chosen. The flow of stimulatory and non‐stimulatory saliva was calculated, and the serums, in addition to salivary levels of aquaporin 5, were determined.

**Results:**

The level of Aquaporin 5 in serum and its output in stimulatory, and non‐stimulatory saliva were significantly decreased, the stimulatory and non‐stimulatory saliva flow was reduced, and the degree of xerostomia was significantly higher in the OLP group.

**Conclusions:**

Aquaporin 5 is implicated in OLP patients in several ways such as impaired salivary functioning, xerostomia or dry mouth, diminished repair ability of the mucosal lesion, increased apoptosis, and probable carcinogenesis in this premalignant lesion.

## 1 Introduction

Oral lichen planus (OLP) is an inflammatory disease mediated by T lymphocytes. OLP affects 0.5%–2% of the population, mostly women between the ages of 30 and 60. The exact cause of OLP is still unclear and many factors are thought to play a role in its pathogenesis (Alrashdan et al. 2016; Abdeldayem et al. 2022). It most commonly affects the oral cavity, but other areas such as the skin, scalp, nails, penis, genitals, esophagus, and conjunctival mucosa may also be affected. The World Health Organization classifies OLP as a potentially precancerous disease with risk of malignant transformation. Patient follow‐ups is recommended (Crincoli et al. 2011).

Common complaints among OLP patients include burning sensations, pain, and, in some cases, dry mouth. Research on xerostomia and hyposalivation in patients with OLP is limited, and research to find the cause and pathogenesis of this condition is still ongoing. Since saliva normally provides mechanical, antibacterial and immune protection, decreased salivary secretion accelerates mucosal epithelial degeneration (Agha‐Hosseini et al. 2020).

Aquaporins are a group of small and hydrophobic membrane proteins which have been widely expressed in animals and plants (Verkman 2005). So far, 13 types of aquaporins have been identified in mammals (Aquaporin 0–12). They have been expressed differently in organs and tissues in fluid discharge of absorption as well as in non‐fluid transporter tissues such as the brain, liver, skin, and lipid (Ishibashi et al. 2009). Aquaporins 1, 2, 4, 5, and 8 are primarily water permeable, while Aquaporins 3, 7, 9, and 10, which are called Aquaglyceroporin, transport glycerol and possibly other small solvents (Yasui 2004). One of the notable characteristics of aquaporin channels is their high selectivity and efficiency in transmitting water or permeation of glycerol except for ions and protons (Madeira et al. 2016). In the previous study, the expression of Aquaporin3 in oral epithelium in oral lichen planus was more than the control group. It could be argued that decreased salivation increases the level of expression of the AQP3 gene to compensate for this deficiency and prevent the onset of xerostomia (Agha‐Hosseini et al. 2020).

It has been well recognized that aquaporin 5 which is expressed in salivary glands has been largely localized in the apical membrane of acinar cells. This highlights the physiological importance of aquaporin 5 in transferring water as transcellular (Matsuzaki et al. 2012).

Sjogren's syndrome as with OLP is a common autoimmune disease in women characterized by hypofunction of the salivary glands. It seems that the cause of hypo‐functionality is multifactorial. Other mechanisms involved in it include blockage of muscarinic receptor of acinar cells by antibodies, impaired water transfer through acinar cells because of changes in the number of aquaporins, diminished sensitivity of salivary acinar cells to neurotransmitters (Dawson et al. 2004). While both OLP and Sjögren's syndrome share some clinical features, such as xerostomia and an autoimmune component, the similarities in their etiopathogenesis remain speculative and require further investigation. In the present study, the salivary level and serum level of aquaporin 5 as a biomarker will be measured in the salivary functioning of patients with OLP suffering xerostomia.

## 2 Materials and Methods

Thirty OLP patients with xerostomia were recruited from Oral Medicine Department. The research project was approved by the Ethics Committee of Tehran University of Medical Sciences (IR.TUMS.DENTISTRY.REC.1396.4192) and all methods were performed in accordance with the guidelines and methods policy (Declaration of Helsinki). Additionally, written informed consent was obtained from the patients before participating in the study.

The inclusion criteria were

1. Confirm the diagnosis of OLP based on observation criteria: bilateral lesions with reticular or papular areas, histopathological criteria are band‐like infiltration of inflammatory cells in the surface layer of connective tissue, consisting mainly of T lymphocytes, hydropic degeneration of the epithelial basal layer, no atypia hyperplasia.
2. Subjective assessment: A xerostomia‐related questionnaire was administered to identify patients with xerostomia. Participants were considered positive for xerostomia if they provided at least four positive responses to the questionnaire (see the Supporting Information).
3. Objective assessment: The lipstick or tongue blade test was used as an additional objective measure to confirm xerostomia (Scully and Felix 2005).

The exclusion criteria included lichenoid reaction to drugs, pregnancy, breast‐feeding, and systemic or local diseases. Further, the patients who had similar lesions in proximity of amalgam restorations were also excluded. Since xerostomia is also observed in diseases such as hypothyroidism, diabetes, anemia caused by iron deficiency, anemia caused by Vitamin B_12_ deficiency, hepatitis, Sjogren's, consuming drugs associated with diminished salivary functioning (anticholinergics, antihistamines, antihypertensive agents, anti‐Parkinson agents, antiepileptic agents, cytotoxic agents, sedative and hypnotic agents, their rejection has been emphasized in this study (Scully and Felix 2005).

Thirty healthy individuals without any systemic disease or drug consumption matched the patients' group in terms of age and gender from among the companions of patients who had referred to the oral and maxillofacial Department. After acquiring written informed consent, they were chosen for taking stimulatory, non‐stimulatory saliva and serum samples.

To rule out diseases causing xerostomia, which were mentioned above, for all patients, CBC, SFe, ferritin, zinc, B12, folic acid, TSH, T_3_, T_4_, FBS as well as HCV Ab, and HBS Ag tests were performed. Sjogren's disease here was ruled out according to the classification criteria presented by American Rheumatology Association in 2012 (ACR) (Shiboski et al. 2012). Further, questions were asked about the consumption of the mentioned drugs to ensure xerostomia not to be associated with drug consumption in these patients.

Next, using a questionnaire related to the symptoms associated with xerostomia, the individuals with this condition were identified as having xerostomia based on at least four positive responses to the questions. Further, the next questionnaire was filled by both groups to determine the intensity of mouth dryness. Every response was classified as follows: never = 1, rarely = 2, sometimes = 3, often = 4, and always = 5. The degree of responses was summed up to the other which resulted in a xerostomia inventory (XI). XI range was as: (1*11) 11 to (5*11) 55 (Thomson and Williams 2000; Torres et al. 2002) (Supporting Information).

All saliva samples were collected in a quiet environment at 10 and 12 o'clock, taking into account the circadian cycle of saliva. Saliva was collected while the patient was fasting, thirsty and at rest for 90 min. The sample was collected in a plastic bottle by the spitting method. To do this, they first swallow the saliva in their mouths and tilt their heads forward with less body and head movement. They put the saliva they collect in their mouths into bottles with numbers and phone numbers on them.

To collect stimulatory saliva, the patients chewed identical pieces of paraffin in terms of shape and weight for 1 min. Then, after expelling the chewing gum out and swallowing the saliva in their mouth, they poured the rest of their saliva into the vial. Eventually, 5 mL of blood was also taken. Next, the samples were centrifuged at 2500*g*, 10 min. The saliva flow was calculated by dividing the level of saliva (mL) by the time (min).

### 2.1 Analysis of Saliva

The serum and salivary level of aquaporin 5 was determined using an ELISA kit (Bioassay Technology Laboratory, China) according to the manufacturer's instructions. After the preparation of the reactants, standard solutions, and samples, 40 mcL of each sample was added to the wells. Next, 10 mcl of Anti‐AQP5 antibody was added to the samples, and eventually 50 mcL of streptavidin‐HRP was added. After coating the plates with a sealer, they were exposed to 37°C for 60 min. After removing the sealer, the plates were washed with wash buffer five times. Once the washing was completed, 50 mcL of substrate of solution A and then 50 mcL of substrate of solution B were added to each well and exposed to 37°C for 10 min. At this stage, 50 mcL of stop solution was added to each well, whereby the blue color quickly turned yellow. Further, 10 min after adding the stop solution, optical density of each well was determined through a microplate reader at 450 nm.

### 2.2 Statistical Analysis

The AQP5 level, which is a quantitative variable, was reported as mean and standard deviation, and independent t‐test was used to compare between the two groups. For the non‐normal distribution data, non‐parametric tests (Mann–Whitney *U* test) were used.

## 3 Results

The OLP patients' group consisted of 30 individuals with the mean age of 54.23 ± 11.93 (minimum: 29, maximum: 77). The control group consisted of 30 subjects with the mean age of 41.40 ± 8.46 (minimum: 27, maximum: 57), chosen from the companions of patients referring to oral and maxillofacial department. The OLP group consisted of 14 male (46.7%) and 16 female (53.3%) patients. The control group consisted of 12 males (40%) and 18 females (60%). The OLP group is significantly older than the control group and gender distribution is balanced between the two groups and no significant difference was observed.

The level of Aquaporin 5 in stimulatory, non‐stimulatory saliva and serum, the Aquaporin 5 output in stimulatory, non‐stimulatory, and serum are provided in Table 1 as Mean ± SEM. The stimulatory and non‐stimulatory saliva flow rates are provided in Table 2 as mean ± SEM. The degree of xerostomia has been reported in Table 2 as median ± IQR.

**Table 1:** Aquaporin 5 in stimulatory, non‐stimulatory saliva and serum.

	Control	Case	*p*‐value
AQP5 in serum (ng/mL)	1.34 ± 5.79	0.84 ± 1.74	0.011*
AQP5 in unstimulated saliva (ng/mL)	0.32 ± 1.09	0.22 ± 0.92	0.653
AQP5 in stimulated saliva (ng/mL)	0.15 ± 0.77	0.02 ± 0.63	0.653
Output AQP5 in unstimulated saliva (ng/min)	0.42 ± 0.20	0.30 ± 0.21	0.047*
Output AQP5 in stimulated saliva (ng/min)	0.49 ± 0.32	0.90 ± 0.33	0.001*

* *p*‐value < 0.05.

**Table 2:** The stimulatory and non‐stimulatory saliva flow rates and the degree of xerostomia.

	Control	Case	*p*‐value
Unstimulated saliva flow (mL/min)	0.75 ± 0.25	0.47 ± 0.26	0.000*
Stimulated saliva flow (mL/min)	1.44 ± 0.45	0.78 ± 0.45	0.000*
Xerostomia (score)	16.00 ± 2.84	30.90 ± 6.98	0.000*

* *p*‐value < 0.05.

## 4 Discussion

Aquaporin 5 (AQP5) plays a critical role in salivary gland function, and its dysregulation has been implicated in various autoimmune and inflammatory conditions. In this study, we observed a significant reduction in AQP5 levels in both serum and saliva of patients with OLP compared to healthy controls. This finding aligns with the clinical presentation of xerostomia in OLP patients, suggesting a potential role for AQP5 in the pathogenesis of salivary gland dysfunction in this condition.

Our results indicate that diminished AQP5 levels may contribute to the reduced salivary flow observed in OLP patients. AQP5 is primarily localized in the apical membrane of salivary gland acinar cells, where it facilitates transcellular water transport. Its under‐expression could impair water secretion, leading to the sensation of dry mouth and other symptoms associated with xerostomia. This mechanism is consistent with findings in Sjögren's syndrome, another autoimmune disorder characterized by salivary gland hypofunction and AQP5 dysregulation (Delporte et al. 2016; Direito et al. 2016; Adhikary et al. 2022). However, unlike Sjögren's syndrome, the role of AQP5 in OLP has not been extensively studied, and our findings provide new insights into the potential mechanisms underlying xerostomia in OLP.

Furthermore, the reduction in AQP5 levels may also impact mucosal healing and epithelial integrity in OLP patients. AQP5 has been shown to play a role in cell hydration, proliferation, and antiapoptotic processes. Its under‐expression could exacerbate epithelial damage and delay mucosal repair, contributing to the chronic nature of OLP lesions (Direito et al. 2016). Future studies should investigate the relationship between AQP5 levels and the severity of mucosal lesions to better understand its role in disease progression.

The presence of autoantibodies against AQP5 or related receptors, such as the M3 muscarinic receptor, could further explain the observed reduction in AQP5 levels. In Sjögren's syndrome, autoantibodies against M3 receptors disrupt AQP5 translocation to the cell membrane, impairing water transport. A similar mechanism may be at play in OLP, as previous studies have reported reduced M3 receptor levels in OLP patients (Agha‐Hosseini et al. 2016). Investigating the presence of anti‐AQP5 autoantibodies in OLP could provide valuable insights into the etiopathogenesis of this condition and identify potential therapeutic targets.

In some tumors, a positive correlation has been developed between the expression of AQPs and histological degree, potential metastasis, and cancer prognosis. Overexpression of AQP3 and AQP5 has been reported in gastric carcinoma, where the level of expression has correlation with metastasis of lymph nodes and lymphatic vascular aggression. Underexpression of AQPs for AQP8 has been observed in colorectal cancer, AQP8 and AQP9 in hepatocellular carcinoma, AQP4 in pleural mesothelioma, and AQP1 in cervical cancer. Therefore, both the reduction and elevation of AQP5 can be associated with the course of carcinogenesis (Wei et al. 2019).

While AQP5 dysregulation has been linked to carcinogenesis in other contexts, our study did not directly investigate this association in OLP. Therefore, any conclusions regarding the role of AQP5 in malignant transformation in OLP would be speculative and require further investigation.

Recent studies have indicated that downregulation of AQP5 is associated with increased apoptotic course of tissues. Since OLP has pathogenesis based on epithelial apoptosis, our results also confirmed this association. Indeed, with AQP5 under expression, its antiapoptotic effects also diminish, which will be in favor of course of OLP (Zhang et al. 2019).

## 5 Conclusion

In conclusion, our study demonstrates that AQP5 levels are significantly reduced in the serum and saliva of OLP patients, which may contribute to salivary gland dysfunction, xerostomia, and impaired mucosal healing in this condition. These findings highlight the potential role of AQP5 in the pathogenesis of OLP and suggest that its dysregulation could be a key factor in the development of dry mouth symptoms. Further research is needed to explore the mechanisms underlying AQP5 reduction, including the potential involvement of autoantibodies, and to evaluate its therapeutic potential in managing OLP. However, conclusions regarding the role of AQP5 in carcinogenesis are beyond the scope of this study and should be addressed in future investigations.

## Author Contributions

Farzaneh Agha‐Hosseini contributed to the conception and design, drafted the manuscript, and gave final approval. Yalda Elham contributed to the acquisition and interpretation, drafted the manuscript, and gave final approval. Iraj Mirzaei‐Dizgah contributed to the analysis and interpretation, drafted the manuscript, and gave final approval. Mahdieh‐Sadat Moosavi contributed to the conception and design, contributed to the interpretation, drafted the manuscript, and gave final approval.

## Conflicts of Interest

The authors declare no conflicts of interest.

## Supporting information

Supporting information.

## Data Availability

All data generated or analyzed during this study are included in this published article.
