# Supplementary material for: Decreased Serum and Salivary Levels of Aquaporin 5 in Oral Lichen Planus
Source: Clin Exp Dent Res. 2025 Mar 18;11(1):e70107. doi: 10.1002/cre2.70107 (PMC11917382; doi:10.1002/cre2.70107)
Supplement: Supplementary file 1 — Supporting information. [file CRE2-11-e70107-s001.docx]

**Questionnaire 1.** Questionnaire used for selection of subjects with xerostomia. Response options: yes/no.

1. Does your mouth feel dry when eating a meal?
2. Do you have difficulties swallowing any foods?
3. Do you need to sip liquids to aid in swallowing dry foods?
4. Does the amount of saliva in your mouth seem to be reduced most of the time?
5. Does your mouth feel dry at night or on waking?
6. Does your mouth feel dry during the daytime?
7. Do you chew gum or use candy to relieve oral dryness?
8. Do you usually wake up thirsty at night?
9. Do you have problems in tasting food?
10. Does your tongue burn?

**Questionnaire 2.** The Xerostomia Inventory (XI). Response options: never (score of 1), hardly (2), occasionally (3), fairly often (4), very often (5).

1. I sip liquids to help swallow food.
2. My mouth feels dry when eating a meal.
3. I get up at night to drink.
4. My mouth feels dry.
5. I have difficulty in eating dry foods.
6. I suck sweets or cough lollies to relieve dry mouth.
7. I have difficulties swallowing certain foods.
8. The skin of my face feels dry.
9. My eyes feel dry.
10. My lips feel dry.
11. The inside of my nose feels dry.
